# Case Report: Catatonia Associated With Post-traumatic Stress Disorder

**DOI:** 10.3389/fpsyt.2021.740436

**Published:** 2021-12-07

**Authors:** Gellan K. Ahmed, Khaled Elbeh, Ahmed A. Karim, Eman M. Khedr

**Affiliations:** ^1^Department of Neurology and Psychiatry, Assiut University, Assiut, Egypt; ^2^Department of Child and Adolescent Psychiatry, Institute of Psychiatry, Psychology and Neuroscience, King's College London, London, United Kingdom; ^3^Department of Psychiatry and Psychotherapy, University of Tübingen, Tübingen, Germany; ^4^Department of Health Psychology and Neurorehabilitation, SRH Mobile University, Riedlingen, Germany; ^5^Department of Neuroscience, Jacobs University, Bremen, Germany

**Keywords:** resignation syndrome, catatonia, asylum seeking, children, post-traumatic stress disorder

## Abstract

We report here about a 12-year-old female patient who had two life-threatening accidents that led to post-traumatic stress disorder associated with catatonia. She had closed eyes, had urinary and fecal incontinence, and had been in an abnormal position for one and half month. Moreover, she had complications such as dehydration, malunion of the fractured arm, and deformities in hand and foot. After detailed psychiatric examination, neurological assessment, and laboratory investigation, the patient received successful treatment in the form of benzodiazepine injections, intravenous fluid, oral antidepressants, and six sessions of electroconvulsive therapy (ECT). We discuss the pathophysiology of catatonia, which remains elusive, and recommend evaluating catatonic children for any possible trauma during psychiatry assessment.

## Introduction

Catatonia, a bizarre psychomotor disease that can manifest with various motor symptoms and even vegetative instability, is one of the most dramatic medical disorders. Catatonia syndrome can be diagnosed as a specifier for psychiatric disorders (including psychotic disorders, mood disorders, neurodevelopmental disorders, and other mental disorders), due to general medical conditions, or catatonia can be non-other specified in the DSM-5 (Diagnostic and Statistical Manual of Mental Disorders-5th edition) (1). This enables the syndrome to be diagnosed in a wide range of psychiatric disorders (2). In DSM-5, catatonia syndrome is not a specifier for post-traumatic stress disorder.

We present here a child who had two life-threatening accidents within 2 weeks that led to post-traumatic stress disorder associated with catatonia.

Our aim was to illustrate the severe psychiatric symptoms associated with traumatic experience in children and discuss the possible pathophysiological link between PTSD and catatonia.

## Case Presentation

S.S., a 12-year-old school girl, is the third of five children. She came with her father to the psychiatry outpatient clinic at the Assiut University in Egypt. She was in an abnormal position (fetal position as to hold her knees close to her chest with her right arms and her left hand in a closed fist position on her chest), had closed eyes, had urinary and fecal incontinence, and showed no verbal response for one and half month. The history was provided by her father.

Her father was a farmer, 50 years old, and illiterate. He claimed that his daughter had normal physical and mental development and was excellent in her school studies. However, two months ago, Miss S.S. had a fight in her uncle's house about money that Miss S.S had lent to her uncle's wife, who refused to give it back. Thus, the patient started to scream, shout, and say obscene words to her uncle's wife. Her uncle's wife closed the room door to prevent her from escaping. She slapped the patient, hit her several times, and threatened to kill her. Moreover, she held the patient's arm backwards until the patient's arm got fractured. The uncle's wife refused to let the patient go until the patient promised not to tell anyone about what had happened. After that, the patient ran to her home with great fear and pain from her fractured arm. Her father was at home and asked what had happened. The patient kept screaming, “My arm. It is very painful,” and refused to answer any of her father's questions but only repeated the statement “She will kill me.” At the hospital, she had an x-ray image and had cast for her arm fracture.

Afterwards, the patient returned home but refused to leave the house. The patient's family reported that initial symptoms were fear and startle responses on encountering loud stimuli and flashbacks. Fearfully, she kept repeating the statement “I will be killed.” She had nightmares about what had happened and kept asking her father to protect her from her uncle's wife. Two weeks later, the oldest sister asked the patient to come with her to the church to pray together. The patient went with her sister and found some of her friends there. She played with her friends in the garden of the church. However, during her play with her friends, a wild dog attacked them. All her friends ran, but the patient fell on the ground and hit her fractured arm on the ground. She screamed, closed her eye, and took the fetal position to protect herself. The wild dog was barking and stood near her but did not bite her. At the same time, her sister saw what happened from the church window, screamed, and went to rescue the patient. Although the patient was rescued, she kept in this position for days and only got relaxed to a certain degree in a supine position during sleep. The patient ate and drank when her sister put the food and water in her mouth. Also, she had urinary and fecal incontinence.

For 15 days, the patient's father tried to calm her, brought religious men to pray for her, and requested for in-home visits from internal medicine doctors to find out what was wrong. The physicians recommended the father to bring his daughter to the University Clinic for Psychiatry as soon as possible. At the beginning, the father refused and was afraid of mental health stigma. However, he approved this recommendation due to the worsening of her symptoms, as the patient's position did not change even during her sleep time. Moreover, she refused to eat or drink at all in the last 3 days.

At the time of admission, the patient had no verbal response, non-ambulatory, forced closure of both eyes, and had a fetal position. She had enuresis/encopresis and did not respond to pain, cold, or touch. She could not be supported to sit or stand on her feet, and she could not do anything when asked to. At the recommendation of the psychiatry consultants in the emergency unit, the patient was admitted to the inpatient psychiatric clinic for further investigations.

## Physical Examination and Investigations

During hospitalization, her vital signs were normal. She demonstrated hypoactivity with virtually no interaction in the examination. She was selectively mute and unresponsive to simple commands but mumbled incoherently at times. She had general rigidity in her legs, body, and neck. The patient's Bush-Francis Catatonia Rating Scale score (3) was 25. Laboratory investigations were done, including serum electrolytes (Ca, MG, Na, and K), complete blood count, and renal and liver function; all were in the normal range.

The following neurophysiological measurements were conducted: electrocardiogram (ECG), electroencephalogram (EEG), and Malta and Widal test (to exclude brucellosis), and all were normal. X-rays of the upper and lower limb (to evaluate the fractured arm and deformities) were done. It was challenging to have any brain image during admission, especially MRI of the brain, as she was in a difficult fetal position with severe rigidity and cannot lie on supine position as required during MRI. In addition, the patient was in acute clinical morbidity, demanding immediate intervention that required time to be stabilized. At this time, neurological examination was done with no abnormality detected (except rigidity) that helped the examiner to exclude other neurological diseases.

The interprofessional psychiatry team diagnosed her as (first diagnosis) having post-traumatic stress disorder (PTSD) and major depressive disorder, single episode, and severe catatonia that needs further assessment. The diagnoses were based on DSM-5 (1).

The orthopedic doctor was consulted and found malunion in the fractured arm with deformity in the foot and hand due to prolonged fixation in an abnormal position. He recommended physiotherapy sessions and follow-up sessions after her psychiatric symptoms improved.

## Treatment

A Ryle tube was used for feeding and catheterization of the patient was carried out. For 3 days, the patient had a benzodiazepine injection (lorazepam 1 mg/12 h per day with little improvement) to treat catatonia and intravenous fluid to treat dehydration. The father gave written informed consent to provide the patient with ECT sessions (by SPECTRUM 4,000 m device, Manufacturer: Indonesia). The patient received two sessions per week for three consecutive weeks, bilaterally, and the patient had muscle relaxant to avoid bone and muscle injury caused by the fit; thus, motoric seizure activity was not observed due to succinylcholine, but seizure lengths on EEG ranged from a minimum of 26 s to a maximum of 30 s. Also, the patient had oral SSRI (Fluvoxamine) 25 mg once a day. The dose was increased after 4 days to 50 mg as recommended for children and adolescents.

## Outcome And Follow-Up

The patient gradually improved after the third ECT session. She started to communicate, first with yes/no answers and with few words, and then with complete sentences. She began to open her eyes and stayed for few minutes in the supine position. After completing her ECT sessions, she improved remarkably and returned to full functioning except for gait difficulties due to her deformed toes.

After 1 month from admission to the psychiatric unit, her Bush-Francis Catatonia Rating Scale score was 0. She was discharged with complete improvement and had a follow-up psychotherapy session for her post-traumatic disorder. The diagnosis at the time of discharge was PTSD associated with catatonia.

## Discussion and Conclusions

A 12-year-old female patient who had two life-threatening experiences developed a post-traumatic stress disorder with catatonia and severe psychiatric complications such as dehydration, malunion of the fractured arm, deformities in the hand and foot, and urinary and fecal incontinence, and showed no verbal response for one and half month. The patient showed remarkable improvement after receiving treatment in the form of benzodiazepine injections, intravenous fluid, oral antidepressants, and six sessions of electroconvulsive therapy (ECT).

The here reported case study is intriguing for several reasons. In children and adolescents, post-traumatic stress disorder regarding types of trauma, features, and symptoms can be different from those in adults. The DSM-5 criteria for catatonia are present in this case as it fulfilled 4 of the 12 specified criteria, i.e., stupor (no psychomotor activity, no reactivity to the environment), mutism (no or minimal verbal response), negativism (not responding to external stimuli or instructions), and posturing. However, the other eight specified criteria, catalepsy, waxy flexibility, mannerism, stereotypy, agitation, grimacing, echolalia, or echopraxia, were not found in the preceding symptoms (1).

Within an hour, the symptoms could wax and wane. Although some are more common than others, there is no single symptom that can diagnose catatonia. Affective disturbances and behavioral problems (e.g., nudism) are also considered part of the catatonic syndrome by some authors, who rely heavily on Kahlbaum's initial descriptions (4, 5). Finally, when autonomic instability is present, the catatonic syndrome can become malignant, resulting in increased mortality (5, 6). To our knowledge, this is the first case report for catatonia-associated post-traumatic stress disorder in a child who did not have resignation syndrome (RS).

Pediatric catatonia is believed to be a rare condition (7, 8), but challenges in recognition and variability in the presentation may lead to under-diagnosis (9). Catatonia should be treated quickly because failing to prioritize this problem can result in a suboptimal outcome if the patient is started on benzodiazepines or ECT too late (10). For example, a case study of the fatal outcome of pervasive refusal syndrome (PRS) in an 11-year-old girl was published in 2015 (11). In the commentary to this work, another expert argues that, actually, the proper diagnosis, in this case, was not pervasive refusal syndrome but catatonia (12).

Although the pathophysiology of catatonia remains elusive, gamma-aminobutyric acid (GABA) and glutamate are two neurotransmitter systems that have been implicated in this psychiatric disorder. According to neuroimaging findings, motor areas of the frontal and parietal cortex have lower resting-state activity and task activation (2). Moreover, catatonia has been linked to up to 40 different signs and symptoms (5, 13).

To date, only few reports found catatonic features associated with PTSD (cf. Hortenstine & Youssef, 2020). On July 1, 2014, the diagnosis of RS was included in the Swedish version of ICD-10 with classification, ICD-10-SE; F 32.3A (14). Instead, the diagnosis is known as catatonia associated with another mental disorder, as in DSM-5 (1).

This diagnosis was established for 424 refugee children and adolescents (0–20 years) who had been treated from 2003 to June 2005 because of reduced communication, motor skills, and inability to carry out daily routines. These children were classified into two grades. The first grade was as follows: children could show some response when spoken to, could walk with support, could do things when requested, and could be spoon-fed. The second grade was as follows: children who needed a nasogastric tube were also mute and were lying down. They were not moving at all, were hypotonic, and blocked from the environment without any formal or emotional contact with people in their environment. Their eyes were closed all the time. The children did not react to touch, sound, pain, or cold. Most had enuresis and encopresis. Some parents managed to take them on a wheelchair regularly to the toilet (15).

Children with grade 2 of RS reported that a majority belonged to an ethnic or religious minority in their homeland, and almost all were persecuted. All had either experienced violence themselves or had witnessed or heard about violence against close family members. The age of onset of the first symptom of illness was 11.2 years for boys and 11.8 years for girls. For falling into a stupor, the age of onset for both boys and girls was 12.9 years. Girls tended to have depression before entering the stuporous condition, while boys tended to have PTSD first. Most children had one or both parents suffering from mental or severe physical disorders. Remarkably, all children with RS in Sweden were affected when they arrived after being exposed to life-threatening traumas in their homeland. Von Knorring et al. (14) concluded that all children with RS in their sample meet DSM criteria for catatonia, and they recommend conducting trials of benzodiazepine (14).

The children's reactions at the triggering moment were very similar to the described concept of learned helplessness known in many mammals (16); when all hope for safety seems to be lost, in an acute fear/stress situation, the individual went into a catatonic-like state, which was irreversible without intensive care. Another well-known concept among both mammals and birds is the acute fear reaction “freezing” or “play dead reaction,” where the old part of the vagus nerve seems to be involved (17, 18). The neurophysiological mechanisms behind RS should be further investigated.

According to Jaspers' definition of pervasive refusal syndrome, the patients refuse actively and are angry to acts of help and encouragement, and no other psychiatric condition could better account for the symptoms (19). Patients with RS are hypotonic, and according to ICD-10, dissociative stupor includes normal muscle tone, and those with dissociative stupor also usually react to loud noise and touch. Children with RS do not respond to any sensory stimulation, not even pain.

Shorter and Fink have argued that catatonia is associated with fear and alarm triggered by trauma. It has been linked to the animal defense of tonic immobility in a predatory environment (20). In the children described, affective, fear, and severe trauma-related disorders are common (21). The history and outcome of the girl in the present study are in line with such a view. A similar view has also been taken in Australia, where a disorder with the same background has been described and labeled traumatic withdrawal syndrome (22).

Regarding catatonia diagnosis and treatment, a concentrated and deliberate effort is required, starting with a challenge test for benzodiazepines; 1 or 2 mg lorazepam is given, ideally intravenously, following an evaluation with a rating scale of catatonias after a half-hour or an hour. The test is considered significant if symptoms decrease by 50%, but a negative test does not exclude the diagnosis of catatonia. When improvement is observed, scheduled doses of benzodiazepine are prescribed. Higher doses than those used for anxiety or depression may be necessary. Many catatonic patients tolerate higher doses of lorazepam, up to 10–20 mg, surprisingly well and without sedation, implying an atypical response to benzodiazepines and disrupted central GABA metabolism in catatonia. If benzodiazepine treatment fails to provide relief or is not tolerated, an intensive course of ECT should be administered. Bilateral ECT is preferred because it is thought to be a more effective form of ECT, sometimes with initial daily application, up to 10–20 treatments followed by continuation ECT, if needed (10). When it comes to PTSD treatment, trauma-oriented psychotherapy and pharmacotherapy (sertraline and paroxetine) are two of the most commonly used treatment modalities in clinical practice (23). As part of a systematic review on the use of ECT for treating PTSD, it was found that the review recommended using ECT for people with depression (23), but in another review, using ECT was recommended for people with chronic, severe, and refractory PTSD (24).

## Follow-Up of the Patient's Perspective

In a follow-up of a psychiatric interview with the improved patient, she described that she had great fear of death after the first accident and had severe fear when her father mentioned the name of her uncle's wife. She asked her parents to drop her out of school so she will not get out of her home again; thus, she will not see this woman again even by chance. She could not have enough sleep as she had nightmares about being dead or hit again by the same woman. On the other hand, when the dog chased her in the second accident, she fell and thought that the dog would let her go if she does not move or breathe. After that, she described that she does not remember what exactly happened as she remembered seeing her family members and hear their voices but like in a dream.

One of the main limitations of this case report was the absence of MRI of the brain as it is required to exclude organic brain disease.

## Data Availability Statement

The raw data supporting the conclusions of this article will be made available by the authors, without undue reservation.

## Ethics Statement

Ethical review and approval was not required for the study on human participants in accordance with the local legislation and institutional requirements. Written informed consent to participate in this study was provided by the participants' legal guardian/next of kin.

## Author Contributions

GA and EK were part of the care team involved in daily discussions. KE and AK supervised the overall report and care. All authors contributed to the article and approved the submitted version.

## Funding

We acknowledge support by Deutsche Forschungsgemeinschaft and Open Access Fund of University of Tübingen. AK was supported by Deutsche Forschungsgemeinschaft (DFG: 407249047).

## Conflict of Interest

The authors declare that the research was conducted in the absence of any commercial or financial relationships that could be construed as a potential conflict of interest.

## Publisher's Note

All claims expressed in this article are solely those of the authors and do not necessarily represent those of their affiliated organizations, or those of the publisher, the editors and the reviewers. Any product that may be evaluated in this article, or claim that may be made by its manufacturer, is not guaranteed or endorsed by the publisher.
